# Dietary modulation of gut microbiota and functional enzymes in savannah honey bees (*Apis mellifera scutellata* Lepeletier)

**DOI:** 10.1007/s00253-025-13615-x

**Published:** 2025-10-16

**Authors:** Nolwandle N. Khumalo, Linda U. Obi, Abdullahi A. Yusuf, Rasheed A. Adeleke

**Affiliations:** 1 Unit of Environmental Sciences and Management, North-West University, Potchefstroom, South Africa; 2 Social Insects Research Group, Department of Zoology and Entomology, University of Pretoria, Hatfield, South Africa

**Keywords:** Honey bees, Diet, Pollen, Gut microbiome, *Apis mellifera scutellata*

## Abstract

**Abstract:**

Honey bees gather pollen from flowering plants, using it as a vital protein source and, in turn, acquire pollen-associated microbes that interact with their existing gut microbiota. Despite their ecological importance, limited information exists regarding the gut microbiota of African savannah honey bees (*Apis mellifera scutellata* Lepeletier) and how diet and its associated microbial community influence this crucial internal ecosystem. This study aimed to investigate the differences in gut microbiota between wild honey bees collected during the flowering season and microbially depleted honey bees reared under semi-sterile conditions and fed various protein diets. To achieve this, freshly hatched worker bees were maintained in hoarding cages and assigned one of four protein diets: fresh sunflower pollen, casein, sterilised casein, or sterilised pollen. High-throughput DNA metabarcoding was then employed to compare the microbial composition of the honey bee gut across these groups. Our findings revealed that the gut of microbially depleted honey bees exhibited higher species diversity and richness. Conversely, the non-core gut microbial community predominated in wild bees and those fed the different protein diets. Specifically, *Commensalibacter*, *Bartonella*, and *Bifidobacterium* were the most dominant bacterial genera across all treatments. Interestingly, *Gilliamella*, a common core gut bacterium, was undetected, while *Apibacter* was exclusively found in wild honey bees. Furthermore, pollen-associated microbes such as *Devosia* and *Pedobacter* were identified solely in the gut of honey bees fed a pollen diet. Functional predictions of the gut microbial community also indicated the presence of key enzymes such as β-glucosidase, β-galactosidase, pyruvate dehydrogenase and phosphoglycerate mutase, which are crucial for enhancing nutrient absorption, digestion, and carbohydrate metabolism. These results underscore the intricate relationship between honey bees, microbes, and plants, offering valuable insights into how diet and its associated microbial communities could shape the gut microbiota of African honey bees.

**Key points:**

• The non-core gut microbiota dominates the African savannah honey bee

• The type of diet influenced the microbial diversity and community abundance in the honey bee gut

• Key enzymes involved in digestion, nutrition absorption, and carbohydrate metabolism were enhanced in the gut

• Pollen-associated microbes found in the diet present potential avenues for probiotic development to improve honey bee health

**Supplementary Information:**

The online version contains supplementary material available at 10.1007/s00253-025-13615-x.

## Introduction

Honey bees are eusocial insects commonly known for producing honey and are responsible for pollinating crops and plants, thus contributing significantly to global food security and biodiversity in many ecosystems (Potts et al. 2015; Romero et al. 2019; Khan et al. 2020). Like most insects during pollination, honey bees harbour and acquire their gut microbiota through contact with the hive material (e.g., honey comb and brood cells) and pollination environment (e.g., pollen, nectar, and seeds) (Zheng et al. 2018). In addition, these microbes can be acquired through oral trophallaxis, in which newly emerged workers consume bee bread and fermented pollen stored within the hive (Martinson et al. 2012; Powell et al. 2014). Newly emerged worker bees contain few or no gut bacteria upon emergence as adults and are usually colonised by the gut microbiota within the first few days (Martinson et al. 2012; Powell et al. 2014; Kapheim et al. 2015; Kwong and Moran 2016), and usually, between days 4 and 9, the gut microbiota becomes fully established (Martinson et al. 2012; Powell et al. 2014).

The honey bee core gut microbiota is composed of the following phylotypes: *Snodgrassella alvi*, *Gilliamella* (e.g., *Gilliamella apicola* and *Gilliamella apis*), *Bombilactobacillus* (formerly known as *Lactobacillus Firm-4*), which consists of two species (*Bombilactobacillus mellis* and *Bombilactobacillus mellifer*), *Lactobacillus* (formerly known as *Lactobacillus Firm-5*), which includes several species such as *Lactobacillus melliventris* and *Lactobacillus kimbladii* (Zheng et al. 2020), and *Bifidobacterium* (e.g., *Bifidobacterium asteroids* and *Bifidobacterium coryneforme*) (Moran et al. 2012; Vojvodic et al. 2013; Moran 2015; Kwong and Moran 2016; Bonilla-Rosso and Engel 2018). In contrast, non-core members include *Frischella perrara*, *Apibacter adventoris*, *Bartonella apis*, *Bombela apis*, and *Commensalibacter* (Alpha 2.1) (Moran 2015; Kwong and Moran 2016; Bonilla-Rosso and Engel 2018; Raymann and Moran 2018). These microbes play vital roles in food digestion, nutrient absorption, host metabolism, and pathogen defence, all of which are essential for the growth and development of honey bees (Bonilla-Rosso and Engel 2018; Raymann and Moran 2018; Khan et al. 2020). Furthermore, they are known for producing a variety of enzymes that facilitate the breakdown and metabolism of plant polysaccharides, such as pectin, starch, hemicellulose, and glycosides (Quinn et al. 2024). For instance, *G. apicola* has been shown to facilitate the metabolism of pectin, mannose, xylose, and arabinose found in pollen by producing the pectin lyase enzyme, which is crucial for pollen digestion (Ricigliano et al. 2017; Zheng et al. 2019).

Beyond the dominant core gut microbes, honey bees also acquire microbes through their diet, with pollen being a significant source of these microbial communities (Khan et al. 2020; Dharampal et al. 2020; Santorelli et al. 2023). Pollen serves as the primary protein source for adult honey bees, supplying essential macronutrients (e.g., lipids, carbohydrates) and micronutrients (e.g., essential oils, vitamins) (Di Pasquale et al. 2013; Geldert et al. 2020; Li et al. 2022). Moreover, pollen contributes to several critical aspects of honey bee health and development: it aids in the development of hypopharyngeal and mandibular glands in adult worker bees during their first few days of emergence, plays a vital role in larval development, promotes colony growth, and offers protection against bee pathogens (Giacomini et al. 2018; Nicolson et al. 2018; Dharampal et al. 2019; Zheng et al. 2019; Du Rand et al. 2024).

While research on pollen diversity, the nutritional quality of pollen or alternative protein, and the honey bee gut microbiome is extensive (Degrandi-Hoffman et al. 2010; Saraiva et al. 2015; Kwong and Moran 2016; Bonilla-Rosso and Engel 2018; Nicolson et al. 2018; Dharampal et al. 2020; Kešnerová et al. 2020; Powell et al. 2023; Santorelli et al. 2023), the precise nature and extent of how pollen-associated microbes are acquired and interact with existing gut microbes, and their potential effects on the composition and diversity of the gut microbiota, remain largely unexplored. Therefore, understanding the microbial community associated with the honey bee gut could provide insights for developing strategies to improve colony health, enhance immunity, and increase resistance against bee pathogens and environmental stresses (Maruscakova et al. 2020; Motta et al. 2022). This is particularly crucial as global honey bee populations are declining.

Our study specifically focused on *Apis mellifera scutellata* Lepeletier, an African honey bee subspecies. This subspecies displays distinct biological and behavioural characteristics compared to its counterparts in the Northern Hemisphere. Consequently, it exhibits reduced susceptibility to various pathogens, pests, and parasites, a trait attributed to its high genetic diversity (Pirk et al. 2016). Additionally, several factors such as shorter development periods, task allocation, and pheromonal composition have contributed to these differences (Pirk et al. 2016; Nganso et al. 2018; Mumoki et al. 2021; Buttstedt et al. 2023a,b). Beekeeping practices in Africa often involve wild, naturally mated queens, which can influence microbial acquisition patterns. In contrast to well-documented European and American bee species, there is limited information on the gut microbiota and the precise timeline of gut microbiome acquisition in African honey bees. A study by Tola et al. (2020) characterised the adult microbiome but did not explore acquisition or the specific influence of diet. Our experimental design, employing newly emerged workers presumed not yet to have acquired a stable gut microbiome, allows us to directly assess the potential impact of different diets as drivers of gut microbiome composition, thereby providing unique insights into acquisition pathways and microbial sources. Hence, this study aims to provide a comprehensive analysis of the adult gut microbiota of African savannah honey bees (*A. mellifera scutellata*). Furthermore, it seeks to establish how microbes within pollen and protein diets influence the gut microbial diversity and composition of microbially depleted honey bees maintained in hoarding cages.

## Materials and methods

### Pollen collection and preparation

Sunflowers (*Helianthus annuus* L.) were collected in March 2021 during their natural flowering season from a commercial farm situated in Wolmaransstad, North West Province, South Africa (27° 09′ 52.0″ S 25° 53′ 46.9″ E). Fresh pollen was meticulously gathered by brushing it from several flower heads using a paintbrush, a method described by Nicolson et al. (2018). Following collection, the pollen grains were mechanically crushed with a pestle and mortar and then stored until required. To prepare the sterilised pollen treatment, fresh pollen was freeze-dried and then immersed in 70% ethanol. This was followed by drying under UV light for a period of 24–48 h, or until the ethanol had completely evaporated, consistent with the method outlined by Dharampal et al. (2019).

### Honey bee collection and hoarding cage setup

**Fig. 1:**
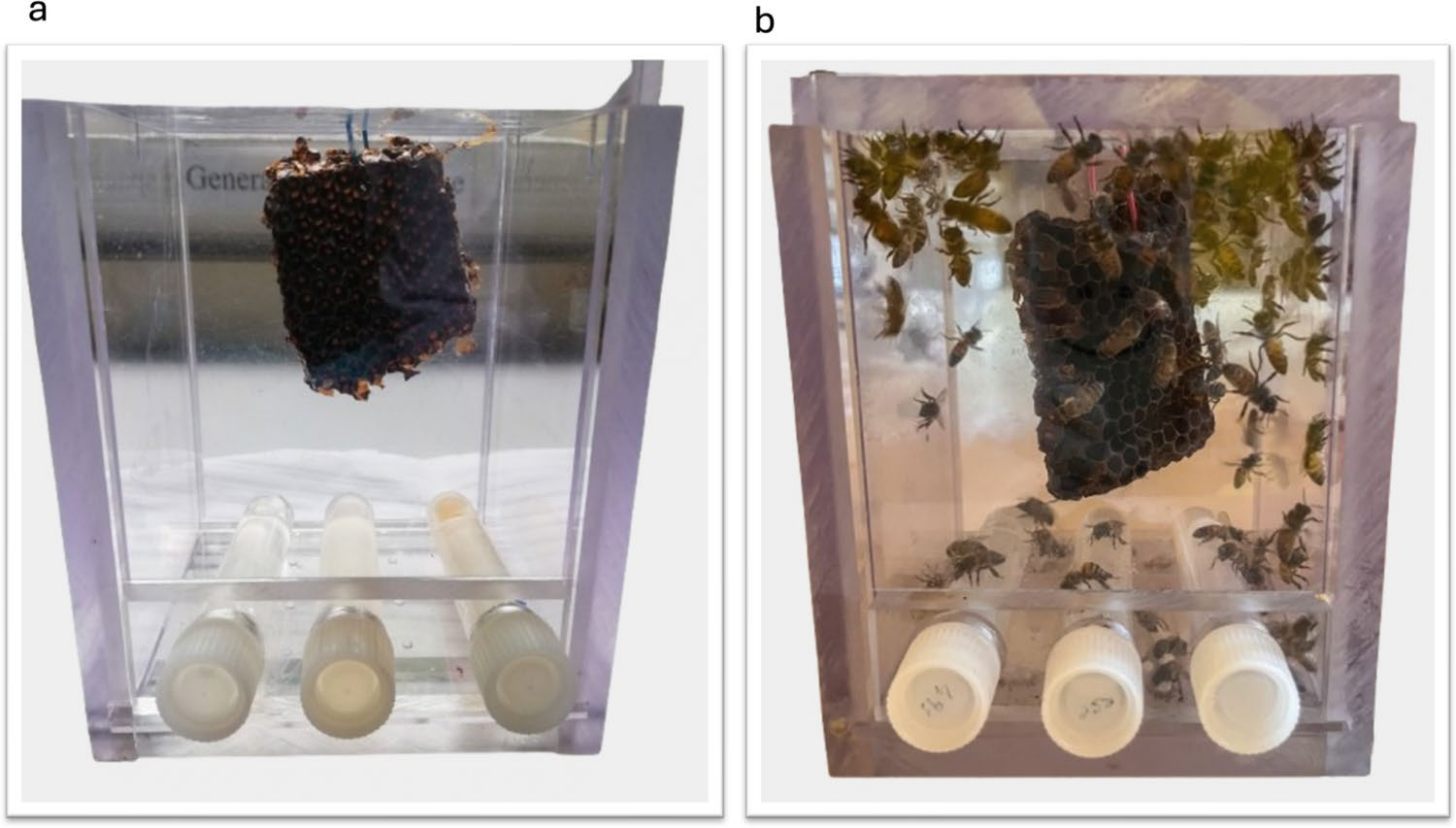
Hoarding cage experiment. **a** A Perspex hoarding cage (120 mm × 95 mm × 80 mm) with two sliding panels, a perforated panel for ventilation at the bottom with three feeding openings, each containing dH_2_0, 30% sucrose solution (w/v), a protein diet, and a piece of honey bee comb, and **b** Each cage contained 100 honey bees and was maintained at 34.5 ºC and 60% relative humidity (RH) for 14 days

Capped worker brood frames were obtained from two colonies of *A. mellifera scutellata*, each led by naturally mated queens, from the apiaries of the University of Pretoria, Gauteng Province, South Africa (25° 45′ 16.1″ S 28° 15′ 26.1″ E)*.* These frames were subsequently incubated under controlled conditions of 34.5 ºC and 60% relative humidity (RH) to accurately simulate the environment within a natural hive. Concurrently, pollen foragers (wild) were collected at the botanical garden of the North-West University, North West Province, South Africa (26° 68′ 23.4″ S 27° 09′ 53.8″ E) during the local flowering period. Upon their emergence from the incubated brood frames, 100 worker bees were carefully placed into hoarding cages (Köhler et al. 2015). These cages had been pre-sterilised by autoclaving at 121 °C and 15 psi for 15 min (Fig. 1). To maintain the integrity of both the honey bee comb and the casein, which served as a protein source, a non-temperature-dependent semi-sterile approach was adopted; both materials were exposed to UV light overnight before being introduced into the hoarding cages. Casein, a protein derived from milk, was specifically employed as an artificial protein substitute and functioned as an experimental control (Du Rand et al. 2024).

### Dietary treatments and experimental monitoring

Each hoarding cage was provided with three feeding tubes. These tubes contained autoclaved distilled water (dH_2_0), a 30% sucrose solution (w/v), and one of four distinct protein treatments: a natural source of protein in the form of fresh sunflower pollen, sterilised pollen, an artificial protein source in the form of casein (Sigma Aldrich, St. Louis, MO, USA), or sterilised casein. Alongside these, a piece of honey comb was also supplied to each cage. A protein-free diet was deliberately excluded from the experimental design, as it was anticipated to severely impair the honey bees’ growth and development. Each of the four treatments was replicated four times, resulting in a total of 16 cages containing 1600 honey bees. Fresh tubes containing the various diets were replenished daily for each cage. Throughout the 14-day experimental period, any dead bees from each cage were removed, and survival rates were computed for each treatment at the conclusion of the experiment. It is noteworthy that at this 14-day age, worker bees typically transition from their in-hive duties to becoming foragers, and their gut microbiota is considered stable and well-established (Kapheim et al. 2015). Finally, on day 14, ten worker bees from each diet treatment were sampled, euthanised on ice and subsequently stored at − 20 °C for downstream analysis.

### Honey bee gut dissections

To prepare for gut dissections, ten worker honey bees were collected from each of four treatment groups: sunflower pollen, sterilised pollen, casein, and sterilised casein, at the conclusion of a 14-day hoarding cage experiment. This resulted in a total of 40 bees per treatment group. Before dissection, bees were thoroughly surface sterilised to eliminate external microbes. Each bee was dipped in 0.1% sodium hypochlorite and 70% ethanol, followed by three washes in autoclaved dH_2_O. Thereafter, abdominal dissection was performed using a standard technique, as illustrated by Carreck et al. (2015). The entire honey bee gut from each treatment, including those collected from the wild, was then placed in a sterile 2 mL microcentrifuge tube containing Phosphate-Buffered Saline (PBS) solution at pH 7.4 and stored at − 20 °C.

### Extraction of genomic DNA from the honey bee gut

A modified DNA extraction procedure, based on the study by Engel et al. (2016), was employed to obtain gut microbiota DNA. Specifically, five honey bee gut samples were collected from each treatment and thawed, after which the PBS solution was discarded. To ensure sample homogeneity and achieve sufficient DNA concentration for sequencing, gut samples from the same treatment were pooled into a single 2 mL ZR BashingBead Lysis tube (0.1 and 0.5 mm) (Zymo Research, Irvine, CA, USA). This pooling was necessary because individual bee gut samples averaged only 26.3 mg, a low biomass that would likely yield an inadequate DNA concentration for sequencing (Taylor et al. 2019). A 3.2 mm steel-chrome bead (Scientific Industries Inc., Bohemia, NY, USA) was added to each pooled gut sample in a Lysis tube containing 180 μL of Buffer ATL (Qiagen, Valencia, CA, USA). Samples were then homogenised at 30 Hz for 5 min using the Qiagen TissueLyser LT. DNA extraction from the lysed samples was carried out using the DNeasy Blood and Tissue Kit (Qiagen, Valencia, CA, USA) according to the manufacturer’s protocol (Rothman et al. 2018). The concentration of the extracted DNA (ng/μL) was subsequently measured using a NanoDrop One Microvolume UV–Vis Spectrophotometer (Thermofisher, Carlsbad, CA, USA). Extracted DNA was stored at − 20 °C until it was sent to the Novogene Sequencing facility (Singapore, Asia) for 16S rRNA sequencing of the V4 hypervariable region.

### 16S rRNA sequencing and library preparation

The 16S rRNA V4 region was specifically selected for amplification. The primers used were 515 F (5′-GTGCCAGCMGCCGCGGTAA-3′) and 806R (5′-GGACTACHVGGGTWTCTAAT-3′) (Zhao et al. 2023), with each forward and reverse primer containing Illumina overhang adapters at the 5′ end (Illumina Inc., San Diego, CA, USA). Each Polymerase Chain Reaction (PCR) product contained 15 μL of Phusion® High-Fidelity PCR Master Mix (New England Biolabs, Ipswich, MA, USA), 0.2 μM of each primer, and 10 ng of target DNA. The thermocycling conditions began with an initial denaturation step at 98 ℃ for 1 min, followed by 30 cycles consisting of 98 ℃ (10 s), 50 ℃ (30 s), and 72 ℃ (30 s), concluding with a final 5 min extension at 72 ℃. Sequencing libraries were generated using the NEBNext® Ultra™ II DNA Library Prep Kit (Cat No. E7645), following the manufacturer’s recommendations. Library quality was assessed using the Qubit® 2.0 Fluorometer (Thermo Scientific, Waltham, MA, USA) and the Agilent Bioanalyzer 2100 system (Agilent, Santa Clara, CA, USA). Finally, the prepared library was sequenced on an Illumina NovaSeq platform (Illumina Inc., San Diego, CA, USA), which produced 250 bp paired-end reads.

### Bioinformatics

Generated sequences were imported into the Qiime2 pipeline (v. 2020.8, https://docs.qiime2.org/2020.8/) as paired-end sequences. The quality of these paired-end sequences was verified using FastQC version 0.12 (Babraham Institute, UK), and low-quality regions at both the 5′ and 3′ ends were subsequently trimmed using Trimommatic software version 0.4 (Bolger et al. 2014). Trimmed sequences underwent denoising, filtering, merging, and clustering into amplicon sequence variants (ASVs) using the DADA2 plugin (Callahan et al. 2016). For high precision, taxonomic classification and arrangement of ASVs were performed by aligning sequences with the BEExact reference database, specifically using the V3–V4 regions of the 16S rRNA genes. BEExact is a specialised, non-redundant database tailored for 16S rRNA gene-based sequencing, designed to analyse honey bee-associated microbial communities (Daisley and Reid 2021). The taxonomic classification of bacterial 16S rRNA gene sequences utilised the BEExact classifier in conjunction with its latest version of the reference database (Daisley and Reid 2021). BEExact facilitates high-resolution taxonomic assignments by integrating all publicly available bee microbiome sequences and resolving phylogenetic inconsistencies through both distance-based and maximum likelihood approaches. Unless otherwise specified, analyses of the gut bacterial community structure were based on 97% rRNA gene sequence similarity. Taxonomic bar plots were generated, and normalised data was applied in the diversity metrics analyses (α and β) using the Phyloseq package in R (v. 4.1.3). Differences between treatment groups in multivariate spaces were evaluated based on Bray–Curtis dissimilarity. The bacterial community structure was visualised in multivariate spaces using non-metric multidimensional scaling (NMDS) and the unweighted pair-group method with arithmetic mean (UPGMA) technique, employing the vegan (v. 2.6–4) and ggdendro (v. 0.2.0) packages of the R software (https://www.r-project.org). The prediction of functional abilities of the 16S rRNA community was performed using the Kyoto Encyclopedia of Genes and Genomes (KEGG; http://www.genome.jp/kegg/) database via Phylogenetic Investigation of Communities by Reconstruction of Unobserved States (PICRUSt2; https://github.com/picrust/picrust2; v. 2.2.0) (Douglas et al. 2019).

### Statistical analyses

All statistical analyses were carried out using R 4.3.1 (R Core Team 2023). For data generated from the dietary trials, normality was assessed using the Shapiro–Wilk test. Since the obtained data were not normally distributed, a non-parametric statistical test, specifically the Kaplan–Meier survival regression, was applied to determine whether diet (casein, pollen, sterilised pollen, and sterilised casein) significantly affected the survival of honeybees over 14 days. The Log-rank (Mantel-Cox) test was subsequently used to ascertain if any significant differences (*p* < 0.05) were observed between the various diet treatments.

## Results

### Survival of honey bees in hoarding cages

**Fig. 2:**
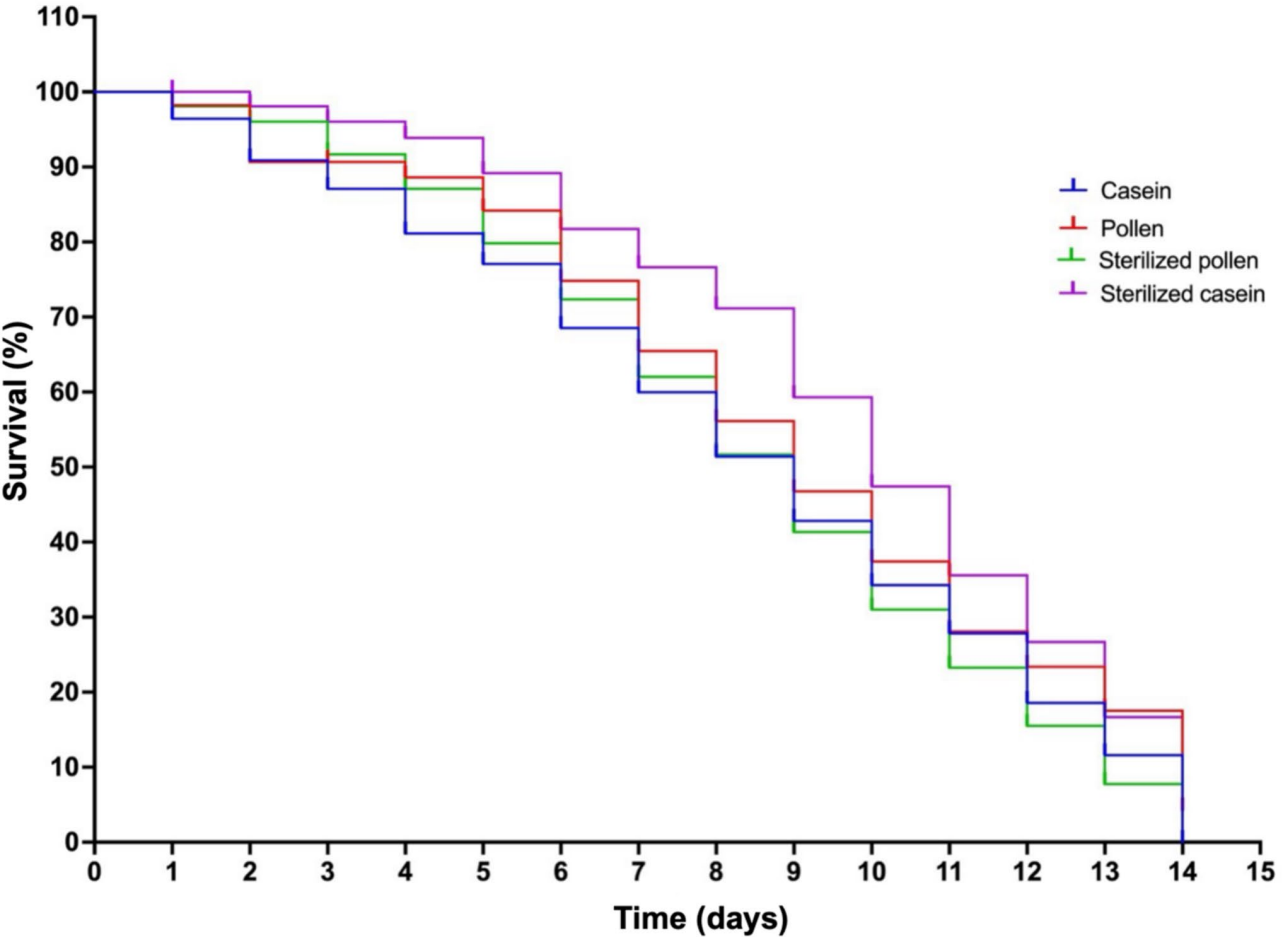
Survival of honey bees in hoarding cages fed artificial protein and natural pollen diets for 14 days. The diets were casein, pollen, sterilised pollen, and sterilised casein

This analysis examines the effects of various diets, namely natural (pollen, sterilised pollen) and artificial (casein, sterilised casein, honeycomb), on the survival of honey bees. Thus, the choice of diet, either natural or artificial, did not significantly affect the survival of honey bees over a 14-day period. This observation is statistically supported (*χ*^2^ = 4.131, df = 3,* p* = 0.2476) and illustrated in Fig. 2.

### Diversity and community structure of honey bee gut microbiota

**Fig. 3:**
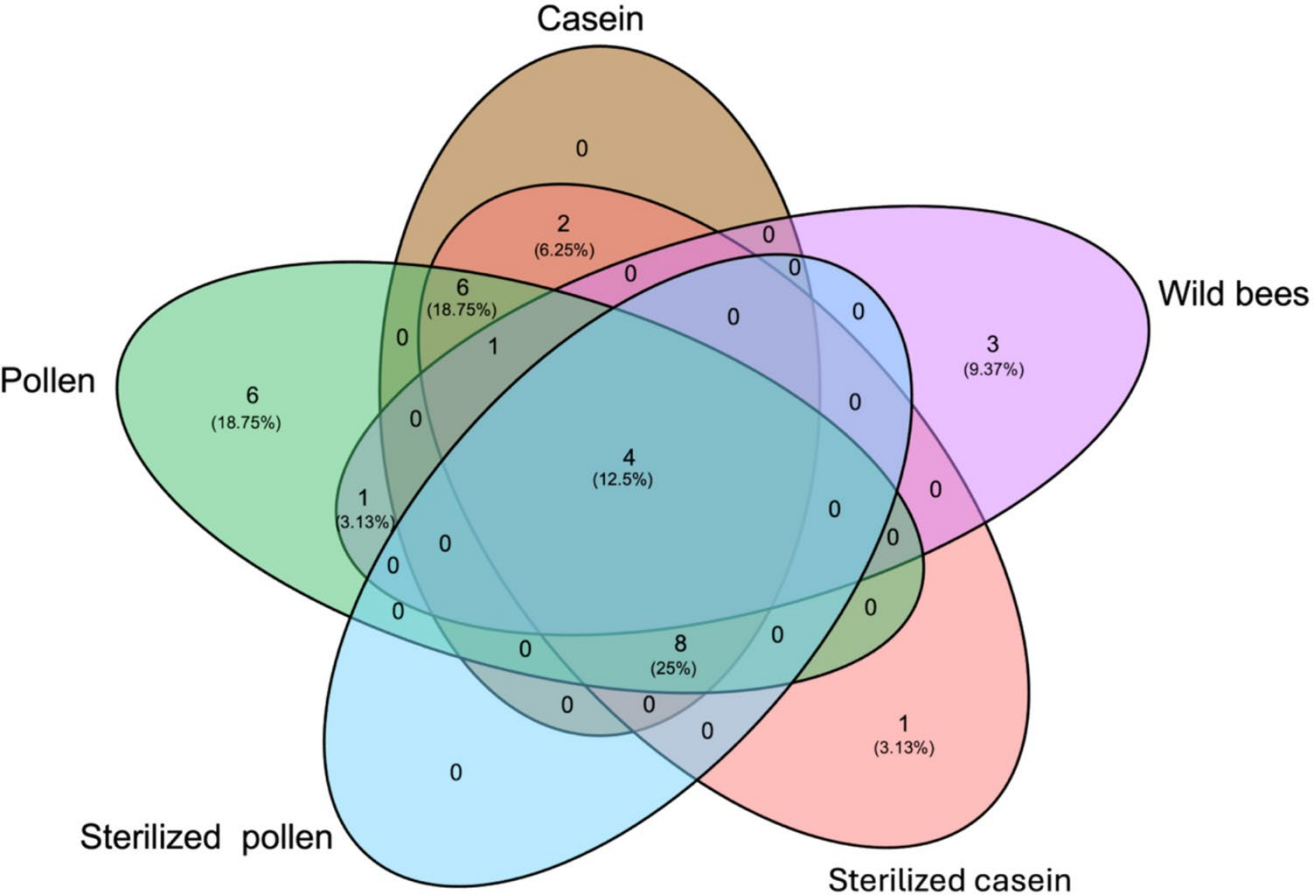
Comparison of unique and shared core amplicon sequence variants (ASV) in the honey bee gut. Adult wild honey bees and those fed different protein diets, namely casein, pollen, sterilised pollen, and sterilised casein

A Venn diagram analysis of the shared and unique ASVs revealed that only 12.5% of amplicon sequence variants (ASVs) were shared across the gut of wild bees and those fed various diet treatments. Honey bees fed pollen exhibited the highest percentage of unique bacterial ASVs (18.75%), indicating a distinct bacterial community, followed by wild honey bees (9.37%) and those fed sterilised casein (3.13%). Intriguingly, the highest percentage of shared bacterial ASVs (25%) was found within the gut of honey bees maintained in hoarding cages under semi-sterile conditions, specifically among those fed casein, sterilised casein, pollen, and sterilised pollen. Following this, a smaller percentage (3.13%) of ASVs was shared between honey bees fed pollen and those collected from the wild. When comparing the impact of protein diet types, honey bees fed an artificial protein source (casein and sterilised casein) shared 6.25% ASVs, whereas honey bees fed a natural pollen diet (pollen and unsterilised pollen) were very distinct, as detailed in Fig. 3 and Supplementary Table S1.

**Fig. 4:**
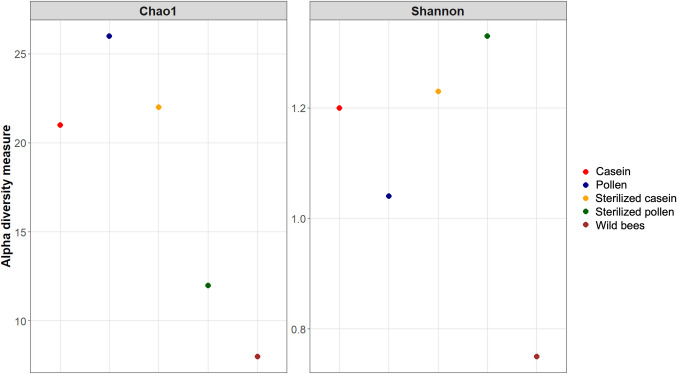
Microbial species richness (Chao1) and diversity (Shannon–Wiener index) in the gut of honey bees fed different diets, namely casein, pollen, sterilised pollen, sterilised casein, and the gut of honey bees obtained from the wild

Honey bees maintained in hoarding cages, particularly those fed pollen, displayed higher species richness, followed by honey bees fed an artificial protein diet (sterilised casein and casein). In contrast, wild honey bees and those fed sterilised pollen showed the lowest species richness. Similarly, species diversity, measured by the Shannon–Wiener index, was the highest in honey bees fed sterilised pollen, while honey bees fed an artificial protein diet demonstrated similar species richness and diversity. Conversely, the gut of wild honey bees exhibited the lowest species diversity compared to all other feed types. These findings are depicted in Fig. 4.

### Feed type and microbial diversity

**Fig. 5:**
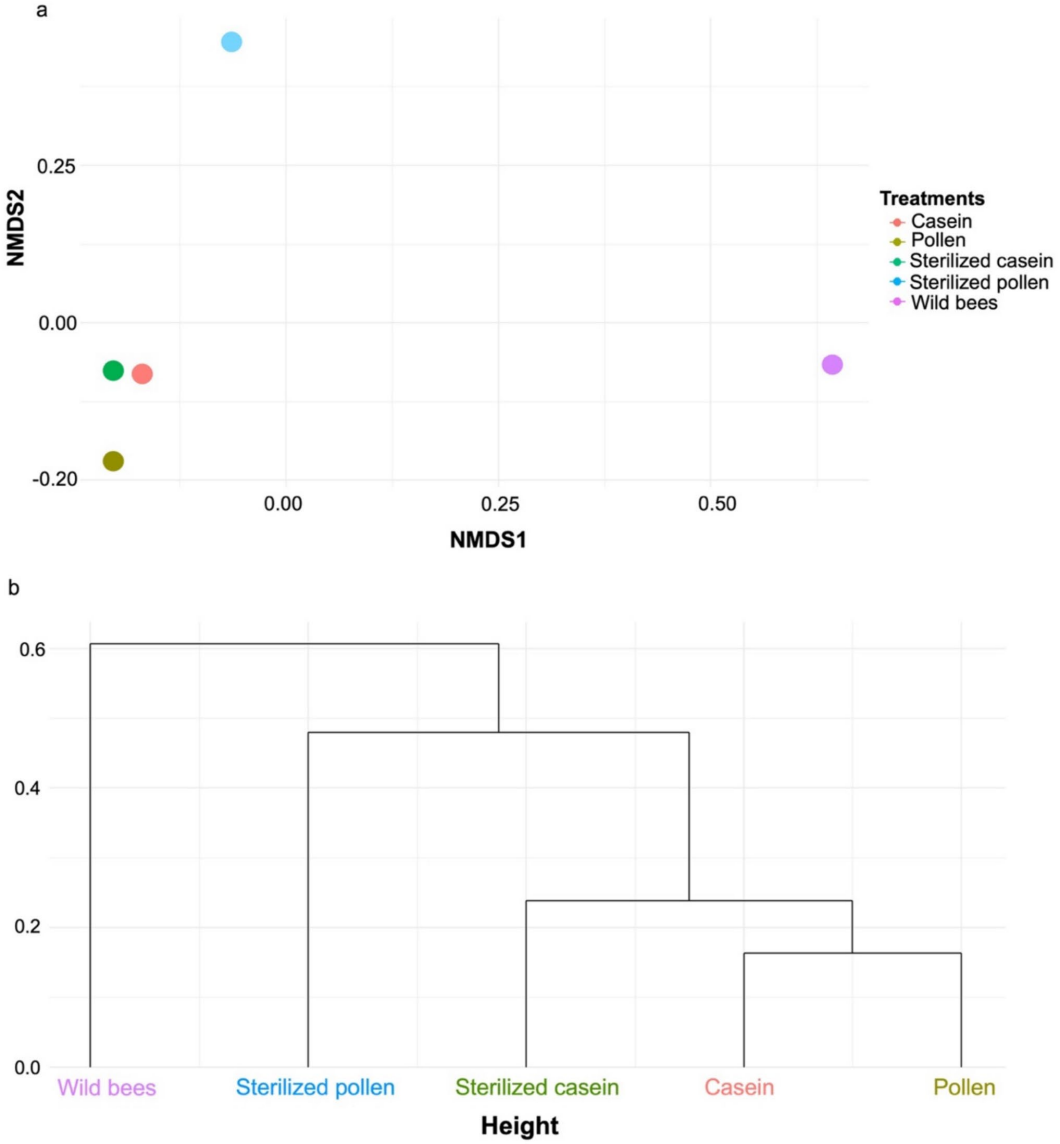
Dissimilarity of honey bee gut bacterial communities fed different protein diets. **a** Comparison of observed ASVs between the gut of honey bees fed casein, pollen, sterilised casein, sterilised pollen, and the gut of honey bees obtained from the wild based on non-metric multidimensional scaling (NMDS), and **b** Unweighted paired group mean arithmetic (UPGMA) hierarchical cluster dendrogram

The Bray–Curtis dissimilarity matrix highlighted a significant shift in microbial composition between the treatment groups. The gut microbiota of wild bees exhibited the most remarkable dissimilarity and clustered separately from the microbially depleted honey bees maintained in hoarding cages. The gut microbiota of honey bees fed an artificial protein diet (casein and sterilised casein) shared a similar bacterial community composition, with the lowest dissimilarity observed (0.20), which suggests that sterilisation of the artificial protein source had minimal impact on the honey bee gut bacterial composition. In contrast, honey bees fed a natural diet (pollen and sterilised pollen) showed a very distinct bacterial community and exhibited the highest dissimilarity (0.59), as shown in Fig. 5a. This observation may suggest that the sterilisation process altered the bacterial community, indirectly emphasising the role of pollen-associated microbes in shaping the honey bee gut microbiota. Furthermore, honey bees fed a sterilised diet (sterilised pollen and sterilised casein) (0.408) also displayed a very distinct bacterial community, indicating that both diet type and its digestibility may play a role in shaping the gut microbiota, even under semi-sterile conditions (Supplementary Table S2). Overall, these results underscore that the gut of wild bees possesses a distinct microbial community compared to honey bees fed either natural or artificial diets, a trend consistently observed in the dendrogram plot (Fig. 5b).

### Bacterial taxonomic community composition in the honey bee gut

**Fig. 6:**
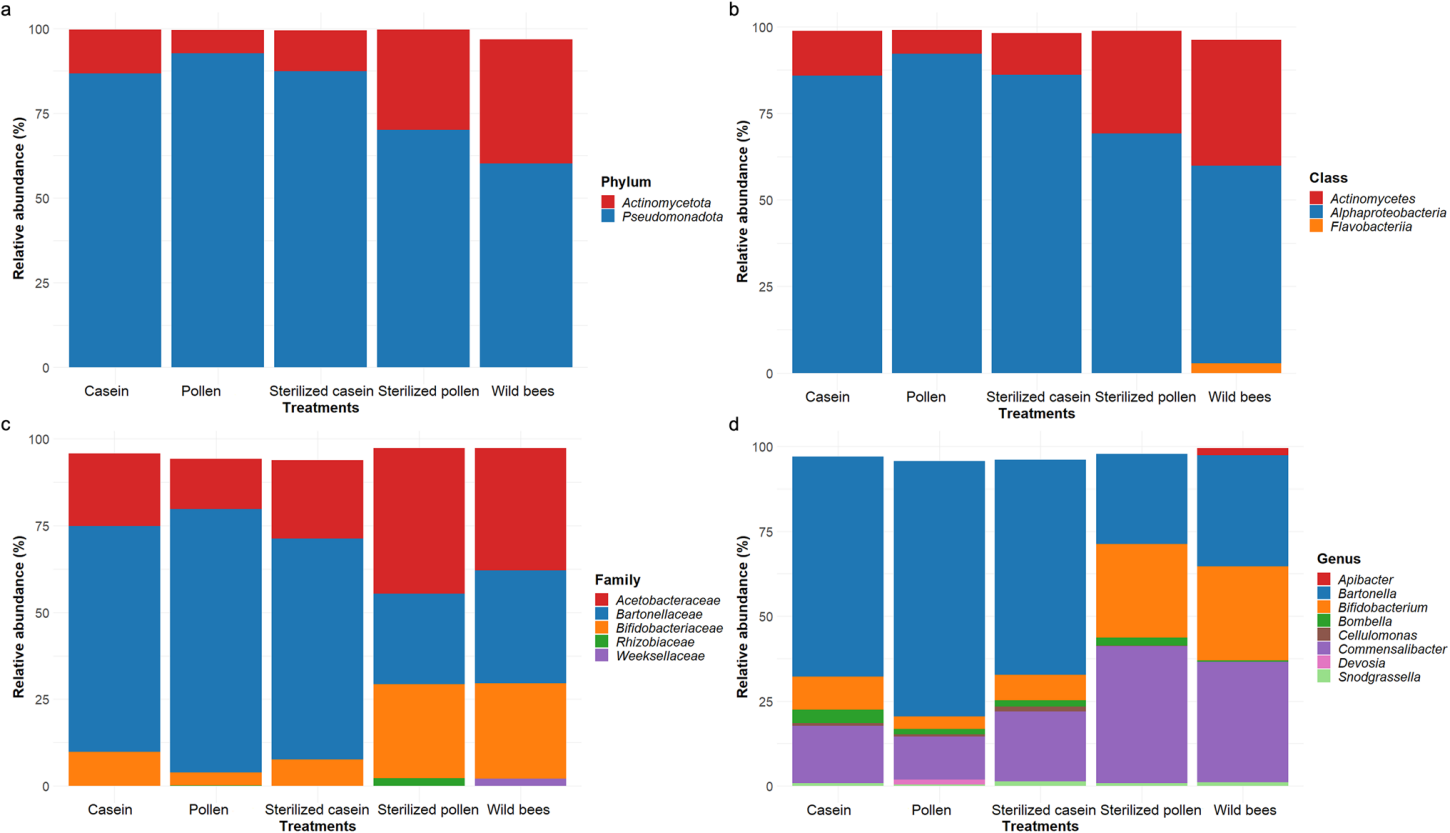
Relative abundance of bacterial community present in the gut of adult wild bees and those fed different diets, namely casein, pollen, sterilised pollen, and sterilised casein. **a** Dominant phyla, **b** Dominant class, **c** Dominant family, and **d** Dominant genus

The honey bee gut microbiota was predominantly composed of the phyla *Pseudomonadota*, followed by *Actinomycetota* (Fig. 6a). Members of the class *Alphaproteobacteria* and *Actinomycetes* constituted the top dominant classes, while the occurrence of *Flavobacteria* was exclusively observed in the gut of wild honey bees (Fig. 6b). At the family level, the most dominant family across all treatments was *Bartonellaceae*, followed by *Acetobacteraceae* and *Bifidobacteriaceae* (Fig. 6c). At the genus taxonomic level, the honey bee gut microbiota was largely dominated by *Bartonella* (52.45%), *Commensalibacter* (25.18%), and *Bifidobacterium* (15.27%), with *Bombella* and *Snodgrassella* present at lower abundances. Notably, the adult honey bee gut was dominated by *Commensalibacter*, *Bartonella*, *Bifidobacterium*, *Apibacter*, *Snodgrassella*, *Bombella*, *Frischella* and *Bombilactobacillus*, while *Bartonella* and *Commensalibacter*, identified as non-core gut bacteria, dominated the gut of honey bees fed different protein diets. Surprisingly, *Gilliamella*, a core gut bacterium, was absent across the various treatments. The highest abundance of *Bartonella* was observed in the gut of honey bees fed pollen, while *Commensalibacter* was most abundant in those fed sterilised casein (Fig. 6d). *Apilactobacillus* and *Lactobacillus* were exclusively present in the gut of honey bees fed different diets. Surprisingly, pollen-associated microbes such as *Devosia* and *Pedobacter* were present only in the gut of honey bees fed pollen (Supplementary Table S3). Furthermore, *Bartonella* and *Bombella* exhibited the highest positive *Z*-scores only in honey bees fed pollen (1.25) and casein (1.69), respectively, indicating their prevalence. In contrast, *Snodgrassella* was more abundant in honey bees fed an artificial diet, specifically sterilised casein (1.00) and casein (0.92). *Bifidobacterium* (1.78), *Bombilactobacillus* (1.79), and *Sanguibacter* (1.77) showed a marked increase in their abundance, but exclusively in the gut of adult wild honey bees (Supplementary Table S4).

### Difference in the honey bee gut microbiota

**Fig. 7:**
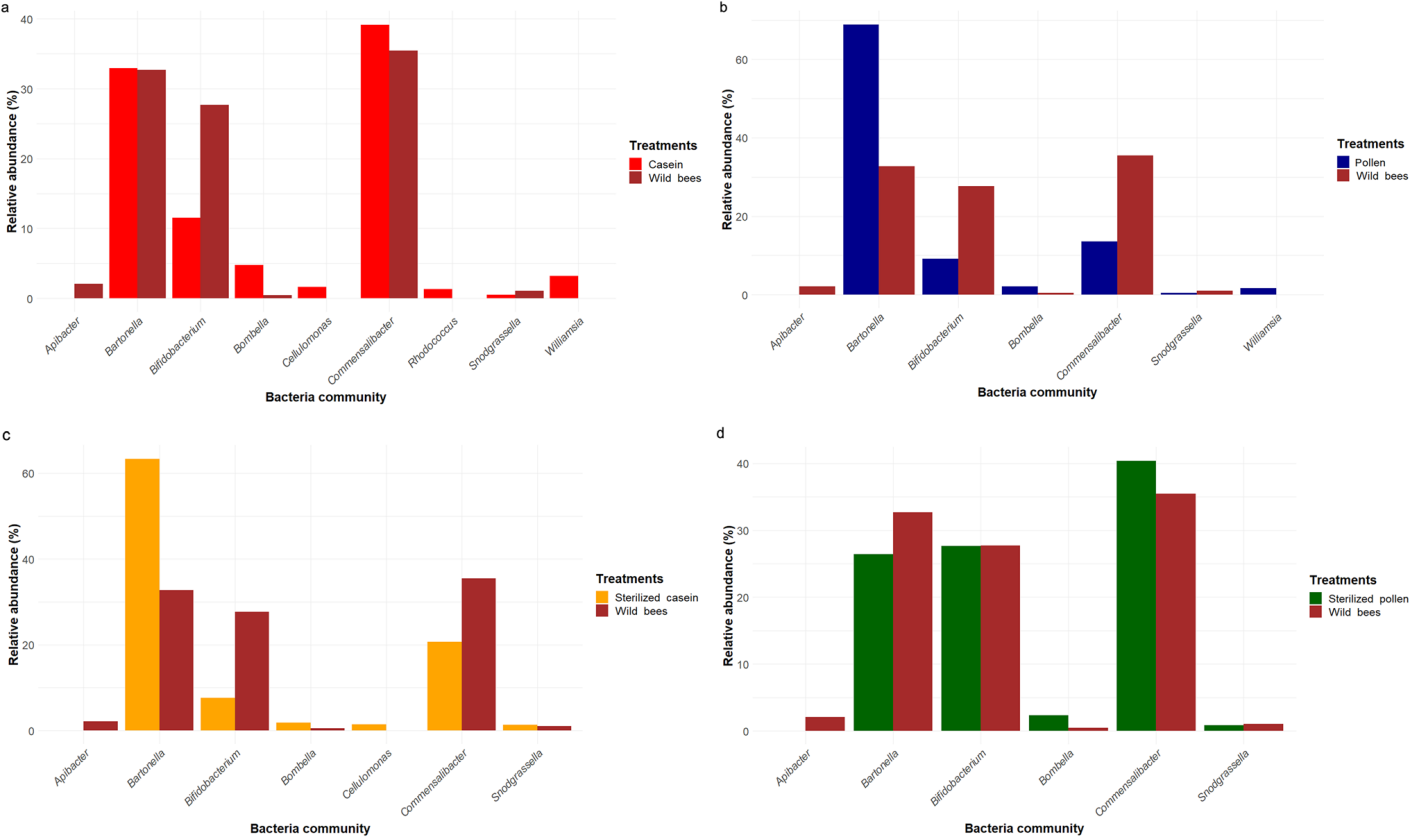
Comparison of the relative abundance of dominant genera in the honey bee gut microbiota of wild bees and honey bees fed different diets at > 1%. **a** Honey bees fed casein, **b** pollen, **c** sterilised casein, and **d** sterilised pollen

A comparison of the bacterial communities in adult wild honey bees and the gut of honey bees fed natural, artificial or microbially reduced diets revealed a higher relative abundance (> 1%) of species comprising the non-core honey bee microbiota, such as *Bartonella* and *Commensalibacter* (Fig. 7). Interestingly, a few species potentially classified as environmental contaminants, transient species, plant-associated species, and opportunistic species, including *Williamsia*, *Rhodococcus*, and *Cellulomonas*, were exclusively found in the gut of honey bees fed different diet treatments. In contrast, *Apibacter* was present only in the gut of wild bees. *Bartonella* exhibited the highest abundance in honey bees fed pollen (Fig. 7b) and sterilised casein (Fig. 7c) when compared to wild bees, while its abundance was similar in honey bees fed casein and wild bees (Fig. 7a). *Commensalibacter* abundance was higher in adult wild honey bees than in those fed pollen and sterilised casein. Moreover, the bacterial composition of the gut of wild honey bees and those fed sterilised pollen was similar, particularly due to the presence of *Bifidobacterium* and *Snodgrassella* (Fig. 7d).

### Predicted functions of the gut-associated microbes

**Fig. 8:**
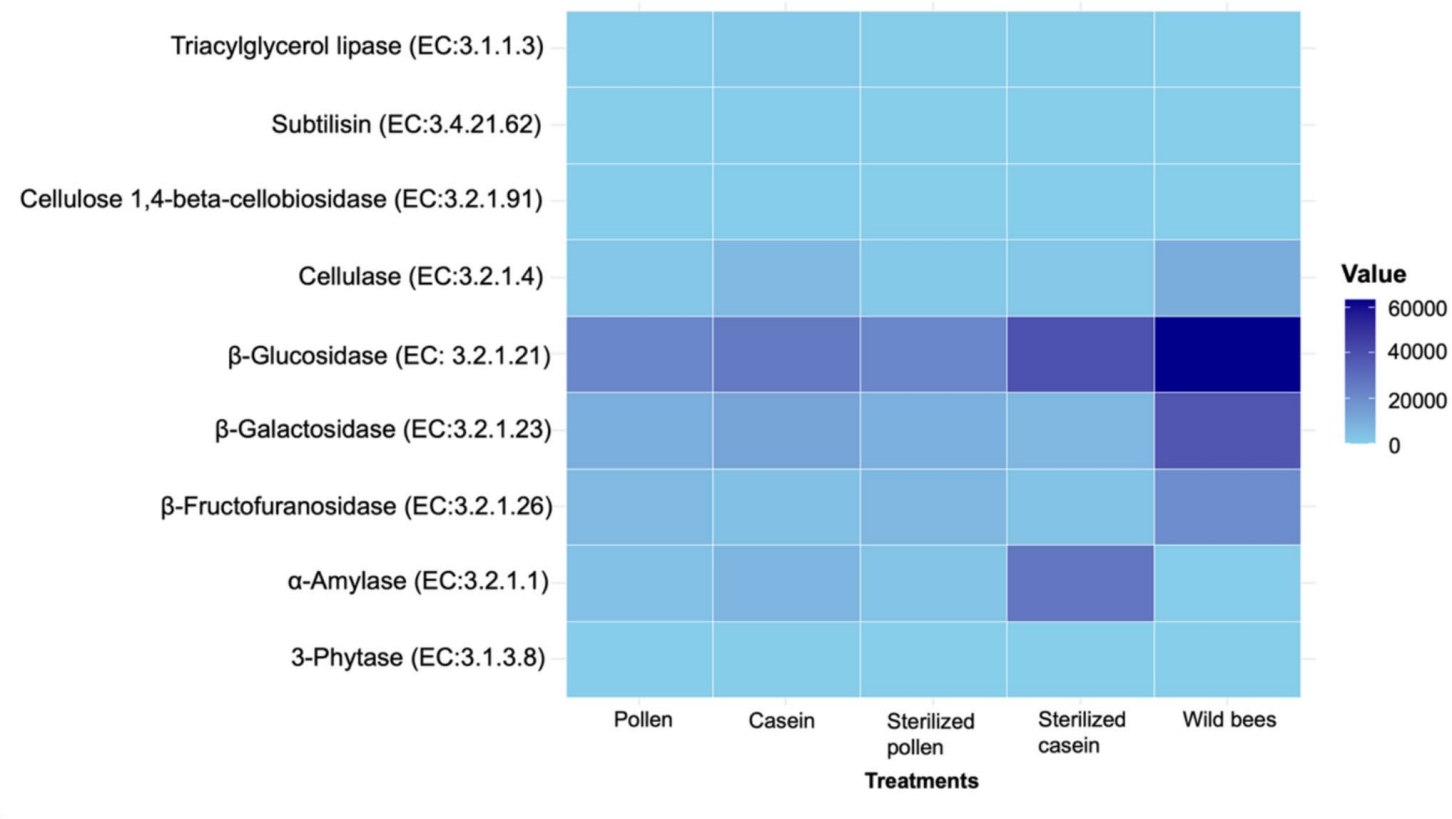
Predicted key enzymes that play a role in improving digestion and nutrient absorption in honey bees. EC numbers were generated from normalised 16S rRNA copy numbers and a set of pre-computed metabolic reference profiles based on the numerical classification system for enzymes. The colour intensity corresponds to the abundance of each gene, with darker shades representing higher abundance values

**Fig. 9:**
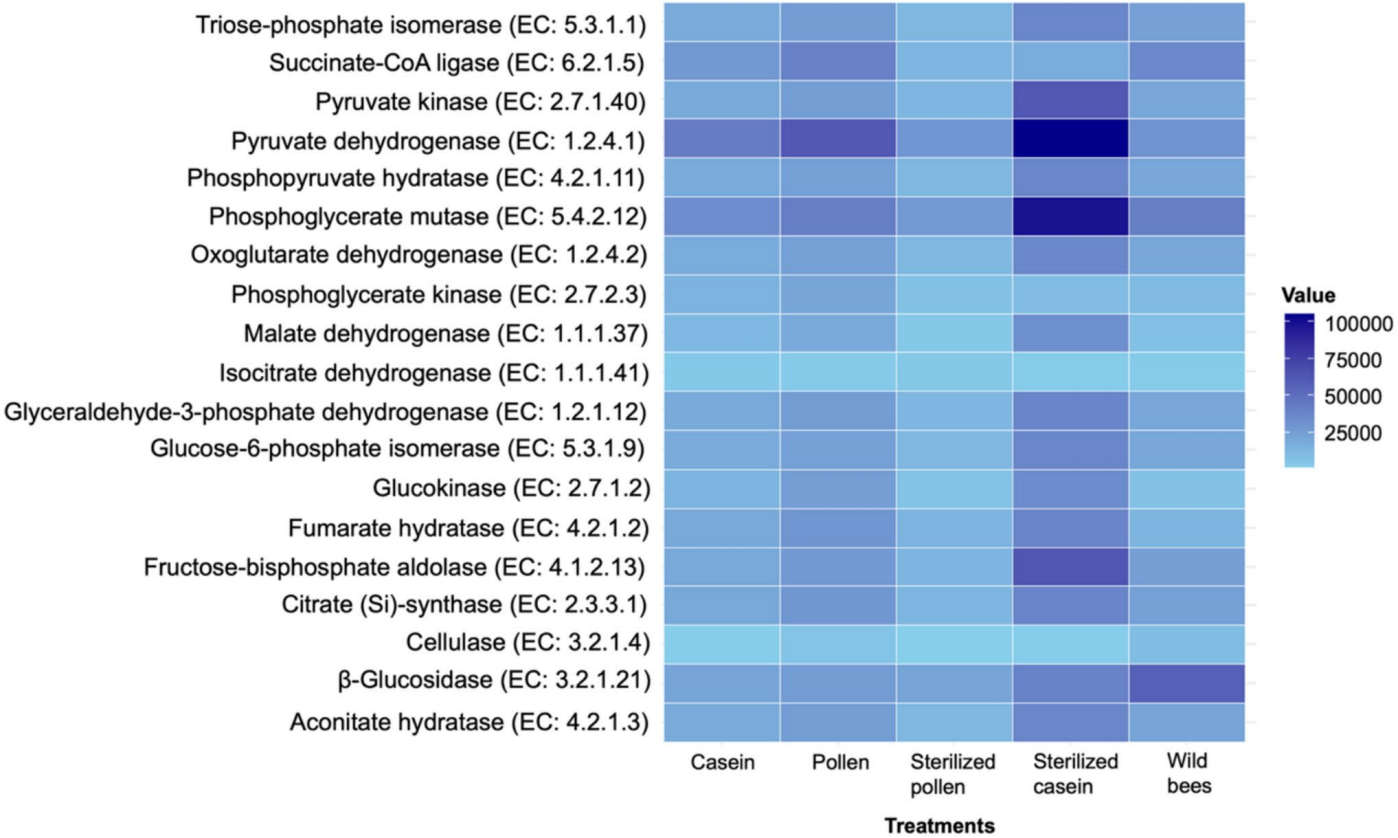
Predicted key enzymes that play a role in carbohydrate metabolism in honey bees. EC numbers were generated from normalised 16S rRNA copy numbers and a set of pre-computed metabolic reference profiles based on the numerical classification system for enzymes. The colour intensity corresponds to the abundance of each gene, with darker shades representing higher abundance values

The functional prediction of key enzymes involved in improving digestion and food absorption revealed several insights. Enzymes such as β-galactosidase, β-glucosidase, β-fructofuranosidase, and cellulase were more abundant in the gut of adult wild honey bees compared to those fed different protein diets (Fig. 8). Notably, the abundance of α-amylase was higher in honey bees fed sterilised casein (Supplementary Table S5). Key enzymes crucial for carbohydrate metabolism, including pyruvate dehydrogenase, phosphoglycerate mutase, and fructose-bisphosphate aldolase, were most abundant in the gut of honey bees fed sterilised casein. Concurrently, β-glucosidase abundance was higher in the adult wild honey bees, as shown in Fig. 9. Surprisingly, the gut of honey bees fed pollen and sterilised pollen exhibited the lowest abundance for most of these digestive and metabolic enzymes (Supplementary Table S6).

## Discussion

The gut microbiota of African savannah honey bees collected from the wild exhibited the lowest species richness and microbial diversity and predominantly featured non-core gut microbiota. Core gut bacteria, such as *Bombilactobacillus*, were found in reduced abundance, and *Gilliamella* was not detected at all. In a hoarding cage experiment, the type of diet, whether natural protein (pollen and sterilised pollen) or artificial protein (casein and sterilised casein), did not impact the survival of honey bees. Microbially depleted honey bees maintained under semi-sterile conditions exhibited the highest microbial diversity, which might be attributed to the digestibility of the protein diet and the sterilisation process. Diet generally increased the relative abundance of non-core gut bacteria across various treatments. Specifically, honey bees fed pollen were colonised by pollen-associated microbes. Wild honey bees, possessing a healthy gut, demonstrated a significantly higher abundance of predicted enzymes crucial for food digestion and nutrient absorption. Conversely, microbially depleted honey bees showed the highest abundance of the predicted enzymes involved in carbohydrate metabolism.

### Honey bee gut microbiota

The honey bee gut microbiota is typically stable compared to other insects and is dominated by approximately 5 to 9 core taxa found consistently in adult bees (Moran et al. 2012; Engel et al. 2016; Kwong and Moran 2016; Bonilla-Rosso and Engel 2018; Zheng et al. 2018). This core community primarily comprises the genera *Gilliamella apicola*, *Snodgrassella alvi*, *Bifidobacterium*, *Lactobacillus*, and *Bombilactobacillus*, which constitute over 95% of the gut microbiota, particularly in European honey bees (Kwong and Moran 2016). However, this composition differs in African honey bees. For example, a recent study by Njoroge et al. (2024) in Kenya found that *Apis mellifera* honey bees were dominated by *Gilliamella*, *Fructobacillus*, *Enterobacter*, *Lactobacillus*, and *Bombella*. In the current study, the *A. mellifera scutellata* honey bees were dominated by non-core gut bacteria such as *Commensalibacter* and *Bartonella*, followed by *Bifidobacterium*, *Apibacter*, *Snodgrassella*, *Bombella*, *Bombilactobacillus*, and *Frischella*. Notably, *Gilliamella*, a core gut bacterium prevalent in Kenyan honey bees was absent across the different treatments, while *Bombilactobacillus*, common in European and North American honey bees (Moran 2015; Kwong and Moran 2016; Bonilla-Rosso and Engel 2018; Tola et al. 2020; Njoroge et al. 2024), was present in extremely low abundance in the gut of wild bees and microbially depleted honey bees across various diet treatments. Interestingly, *Lactobacillus* was exclusively detected in the gut of honey bees that received different diet treatments.

Surprisingly, *Apibacter* was exclusively found in the gut of wild adult honey bees, as opposed to those fed various diet treatments. While Tola et al. (2020) reported *Apibacter* in Kenyan honey bees, most studies have primarily identified this bacterium in honey bees from South and East Asia, including *Apis dorsata* and *Apis cerana*, as well as in bumble bees (Kwong and Moran 2016; Praet et al. 2016; Kwong et al. 2018; Chen et al. 2024). *Apibacter* is a microaerophilic bacterium that colonises the gut wall of honey bees, specifically found in the midgut, ileum, and rectum (Kwong et al. 2018; Zhang et al. 2022). Intriguingly, it occupies the same niche as *Snodgrassella* (Zhang et al. 2022). Its metabolic pathways are involved in the limited breakdown of monosaccharides (glucose, fructose), dicarboxylic acids, and glycerol. However, *Apibacter* may not be involved in the breakdown of complex polysaccharides (Kwong et al. 2018).

Diet has consistently emerged as a primary factor influencing changes in the honey bee gut microbiota, profoundly affecting the abundance and diversity of core gut microbiota. For example, microbially depleted honey bees, kept under semi-sterile conditions, exhibited the highest bacterial diversity (Fig. 3). Their gut also contained numerous transient species, environmental contaminants, and plant-associated microbes. These microbes were present in low abundance compared to the gut of adult wild honey bees (Supplementary Table S3). Their presence in the gut of honey bees fed various treatments could be attributed to sterilisation and contact with the hive environment, including the honey comb and brood frame. For instance, the honey comb placed in the hoarding cages for honey bees fed pollen, casein, and sterilised pollen was not sterilised and potentially harboured hive-associated microbes. Furthermore, the newly emerged bees were in contact with non-sterilised surfaces, such as the brood frames, which may have further introduced environmental microbes. Research indicates that honey bees exposed solely to oral trophallaxis, or comb material, develop an atypical gut microbial community dominated by non-core taxa (Powell et al. 2014). This aligns with the current study’s findings, where non-core gut bacteria like *Bartonella* and *Commensalibacter* dominated the gut microbiota across all tested diet treatments (Fig. 6d).

*Bartonella*, a non-core gut bacterium typically found in low abundance due to its niche restriction and association with the hive rather than the gut environment (Kešnerová et al. 2016; Li et al. 2022), nevertheless dominated the gut microbiota of honey bees across different treatments in this study (Fig. 6d). A comparative genomics study by Segers et al. (2017) revealed that *Bartonella* possesses pathways for breaking down aromatic compounds. These pathways assist in the breakdown of specific pollen components, providing pollen-derived amino acids essential for regulating honey bee labour division, nutrient intake, and physiological metabolism (Fengkui et al. 2015; Segers et al. 2017; Li et al. 2022). Our findings support this, as the abundance of *Bartonella* was highest in honey bees fed pollen (Fig. 6d). Similarly, *Commensalibacter*, another non-core gut bacterium, also dominated the gut of adult wild bees and those on different diets (Fig. 6d). *Bombella* and *Commensalibacter* are acetic acid bacteria of the *Acetobacteraceae* family, known to colonise hive-associated environments such as bee bread, corbicula pollen, nectar, and royal jelly (Corby-Harris et al. 2016; Botero et al. 2023). These acetic acid bacteria provide nutrition to honey bees in sugar-rich environments and are crucial for food digestion, immune response, and defence against pathogens (Corby-Harris et al. 2016; Smith and Newton 2020; Miller et al. 2021).

*Bifidobacterium*, a well-known core gut bacterium typically found in high concentrations in the rectum, was the sole core gut bacterium abundant in this study (Fig. 6d). *Bifidobacterium* is recognised for its ability to digest and metabolise various pollen-associated polysaccharides, including flavonoids, phenolamides, cellulose, pectin, and ω-hydroxy acids from the pollen wall. This highlights its essential role in pollen digestibility and its potential as a significant nutritional source (Kwong et al. 2014; Ricigliano et al. 2017; Bonilla-Rosso and Engel 2018; Zheng et al. 2019; Powell et al. 2023; Kim et al. 2024; Li et al. 2025). Furthermore, *Bifidobacterium* contributes to honey bee health by producing bacteriocins and other antimicrobial compounds that protect against bee pathogens, thereby modulating the honey bee immune system through the synthesis and production of various antimicrobial peptides (Kim et al. 2024; Braglia et al. 2025; Li et al. 2025). Surprisingly, in the present study, honey bees fed pollen exhibited the lowest abundance of *Bifidobacterium*, while its abundance was highest in honey bees fed sterilised casein (Fig. 6d). In addition, *Apilactobacillus*, a lactic acid bacterium, was detected only in the gut of honey bees fed a different diet (Supplementary Table S3). *Apilactobacillus* demonstrates promising potential as a probiotic, capable of reducing the in vitro growth of *Paenibacillus larvae* and the spore load of *Nosema ceranae* (Kiran et al. 2023).

### Artificial protein diets

During periods of pollen scarcity, honey bees are often provided with artificial protein diets designed to mimic the nutritional content and functional characteristics of natural pollen (Degrandi-Hoffman et al. 2010; Zheng et al. 2014; De Jong et al. 2015; Bryś et al. 2021; Ricigliano et al. 2022). These diets typically consist of plant proteins such as soy, yeast, and peas, as well as casein, essential oils, probiotics, organic acids, and various amino acids (e.g., arginine, tryptophan, leucine, histidine) (Di Pasquale et al. 2013). In the present study, casein was utilised as an artificial protein source due to its extensive use in nutritional research (Du Rand et al. 2024). While casein is not a natural component of honey bee diets, it has been observed to positively stimulate colony performance parameters under in vitro conditions (Pirk et al. 2010; Du Rand et al. 2024). Furthermore, artificial protein sources formulated to resemble natural hive conditions have demonstrated positive effects on the gut microbiota of honey bees (Kim et al. 2024). For example, a study by Kim et al. (2024) reported that honey bees fed a pollen substitute diet exhibited increased abundances of *Lactobacillus*, followed by *Rhizobiaceae*, *Snodgrassella*, and *Erwiniaceae*. These bees also showed elevated vitellogenin expression levels compared to a control group. Given that vitellogenin serves as a biomarker for dietary quality and nutritional status, its increased expression suggested a beneficial impact on hypopharyngeal gland development and overall honey bee health (Kim et al. 2024). In the present study, honey bees fed an artificial diet displayed a similar bacterial community composition, primarily dominated by *Bartonella*, *Commensalibacter*, and *Bifidobacterium* (Fig. 6d), and the notable differences observed might stem from the sterilisation of casein and the honey comb. While an artificial diet can match the protein and macronutrient profile of natural pollen, it may still alter the gut’s microbial composition and diversity (Powell et al. 2023). Such shifts or changes in the honey bee gut microbiota can lead to dysbiosis, negatively impacting the honey bee’s immune system, survival, and susceptibility to pathogens (Maes et al. 2016; Zheng et al. 2019; Castelli et al. 2020; Ricigliano et al. 2022; Powell et al. 2023).

### Plant-associated microbes

During foraging, honey bees ingest not only pollen from flowering plants but also the associated microbes. This introduction of new taxa into the honey bee gut can aid in digestion, enhance honey bee growth and development, and bolster their immune system (Braglia et al. 2025; Li et al. 2025). Given pollen’s rigid exine structure and resistance to enzymatic degradation (Kešnerová et al. 2017), honey bees have evolved a symbiotic relationship with plant-associated bacteria that perform crucial hydrolytic activities necessary for complex pollen digestion (Li et al. 2025). For example, a study by Brown et al. (2022) demonstrated that honey bees fed pollen showed enhanced survival rates and increased body weight. Moreover, a greater abundance of pollen-associated microbes in the honey bee gut correlated with higher expression of developmental and immunity genes, including those for vitellogenin and juvenile hormone esterase (Powell et al. 2023). These honey bees also displayed resistance to *Serratia marcescens*, an opportunistic bacterial pathogen, a trait not observed in those fed an artificial diet (Powell et al. 2023). Consequently, diets rich in diverse plant polysaccharides foster a more distinct and diverse gut microbiota compared to pollen substitutes (Powell et al. 2023; Li et al. 2025), positively influencing the microbial composition of the honey bee gut (Ricigliano et al. 2017; Geldert et al. 2020; Ricigliano and Anderson 2020; Powell et al. 2023). In the present study, honey bees fed a natural protein diet namely pollen and sterilised pollen showed the highest species diversity and richness, respectively (Fig. 4). However, a significant limitation of the current study, when compared to research by Powell et al. (2023) and Zumkhawala-Cook et al. (2024), was identified: the microbially depleted honey bees did not have the opportunity to fully acquire and establish their social gut microbiota in hoarding cages before being introduced to the various diet treatments. As a result, it is not possible to definitively ascertain whether the observed differences in the gut microbiota are solely due to diet or if other variables contributed. Nevertheless, our study demonstrates the influence of diet on the gut microbiota in honey bees maintained in hoarding cages under semi-sterile conditions.

Interestingly, the gut of honey bees fed a pollen diet contained specific pollen-associated microbes such as *Devosia*, *Pedobacter*, and *Sanguibacter*. *Cellulomonas* and *Rhizobium* were present in low abundance across all treatments (Supplementary Table S3). These microbes may not form a stable symbiotic relationship with the core gut microbiome; instead, they might be transient colonisers or environmental contaminants acquired through contact with the hive environment (e.g., corbicula pollen, nectar, bee bread) during foraging. While the precise role of these identified microbes in the honey bee gut remains unknown, it is recognised that pollen-associated microbes in plants can function as plant growth-promoting endophytes, colonising various plants including potatoes, maize, strawberries, beans, tomato plants, seeds, oil palm, and rice (Lopez-Lopez et al. 2010; Manter et al. 2010; Cho et al. 2016; Mohd Nor et al. 2017). For example, a study by Chhetri et al. (2022) showed that *Devosia* spp. exhibit plant growth-promoting attributes in vitro, including the production of indole acetic acid (IAA) and the solubilisation of siderophores. Their genome also contains genes for siderophore production, tryptophan biosynthesis, and gene clusters involved in the detoxification of various metal pollutants (Chhetri et al. 2022). This suggests that *Devosia* spp. could facilitate the bioremediation of heavy metals in contaminated environments.

### Sterilised diets

In this study, sterilisation was used to reduce the microbial load and abundance of certain opportunistic and beneficial bacteria in the honey bee gut, allowing for the observation of which specific microbial taxa would dominate under these conditions. Honey bees fed a sterilised diet (sterilised casein and sterilised pollen) showed a significantly higher abundance of *Commensalibacter, Lactobacillus,* and *Snodgrassella* (Supplementary Table S3) compared to those fed unsterilised casein and pollen. In contrast, a study by Li et al. (2025) found that honey bees fed UV-radiated sunflower pollen were dominated by *Lactobacillus*, *Frischella*, *Gilliamella*, *Bifidobacterium*, and *Commensalibacter*. This previous study also reported increased expression of immunity-related genes and higher antioxidant enzyme activity, potentially due to the nutrient content of sunflower pollen (Li et al. 2025).

### Possible functional roles of the gut microbiota

Our genomic inferences indicate that the honey bee gut microbiota harbours key enzymes vital for nutrient absorption, digestion, and carbohydrate metabolism, playing essential roles in host growth, metabolism, and pathogen defence. Our predictions further suggest wild honey bees had a higher abundance of digestive enzymes such as β-galactosidase and β-glucosidase, crucial for pollen breakdown, reflecting a synergistic host-pollen-enzyme relationship (Kunieda et al. 2006; Ricigliano et al. 2017). Conversely, α-amylase, important for starch hydrolysis, was predicted to be more abundant in bees fed sterilised casein. Pollen-associated microbes generally contribute various hydrolytic and detoxifying enzymes, facilitating the breakdown of complex pollen components and plant toxins (Engel et al. 2012; Lee et al. 2015; Zheng et al. 2019; Banerjee et al. 2022; Dong et al. 2023). Enzymes for carbohydrate metabolism, such as pyruvate dehydrogenase, were predicted to be most prevalent in bees fed sterilised casein, while pollen-fed bees were predicted to have lower levels for most digestive and metabolic enzymes. Dominant bacteria such as *Bartonella* (highest in pollen-fed bees) are implicated in pollen aromatic compound breakdown, and *Bifidobacterium* actively digests pollen polysaccharides and supports immunity (Fengkui et al. 2015; Segers et al. 2017; Li et al. 2022, 2025; Kwong et al. 2014; Ricigliano et al. 2017; Bonilla-Rosso and Engel 2018; Zheng et al. 2019; Powell et al. 2023; Kim et al. 2024; Braglia et al. 2025). Additionally, *Apibacter* was exclusively found in wild bees, while pollen-associated *Devosia* and *Pedobacter* (present only in pollen-fed bees) may contribute plant growth-promoting traits, though their specific gut roles are unclear (Kwong et al. 2018; Chhetri et al. 2022).

## Conclusion and recommendations

This study provides significant insights into the gut microbiota of the African savannah honey bee (*A. mellifera scutellata*) from South Africa. It reveals the presence of both core and non-core bacterial communities, with notable differences compared to previously described European and North American honey bees. A key finding was the dominance of non-core gut bacteria, specifically *Commensalibacter* and *Bartonella*, across all tested treatments, including wild bees. Interestingly, *Apibacter* was exclusively detected in the gut of wild adult honey bees, while *Gilliamella*, a typical core gut bacterium, was largely undetected across all treatments. These findings suggest that the protein source—whether natural pollen, artificial casein, or sterilised variants—could influence the gut microbial community of microbially depleted honey bees maintained under semi-sterile conditions.

Pollen-based diets fostered the abundance of taxa implicated in complex pollen digestion and introduced microbes originating from the pollination environment, directly suggesting that honey bees may acquire and harbour microbes associated with their dietary intake. The observed shifts in the gut microbial community correlated with predictions of key enzyme abundances crucial for carbohydrate metabolism, nutrient absorption, and digestion. Wild honey bees exhibited a significantly higher abundance of predicted enzymes involved in food digestion and nutrient absorption, such as β-galactosidase and β-glucosidase, compared to diet-fed bees. Conversely, microbially depleted honey bees fed sterilised casein showed the highest abundance of predicted enzymes involved in carbohydrate metabolism, such as pyruvate dehydrogenase. Notably, despite its role in pollen digestion, *Bifidobacterium* abundance was the lowest in pollen-fed bees and the highest in sterilised casein-fed bees.

In summary, this study demonstrates how diet could serve as a critical determinant of honey bee gut microbial diversity, composition, and functional capabilities, thereby influencing digestion, nutrient uptake, and potentially survival. We acknowledge inherent limitations, such as the potential for environmental microbial influx from UV-sterilised hive components (e.g., honey comb) and the fact that experimental honey bees did not fully acquire a stable social gut microbiota before treatment initiation. Nonetheless, our experimental design enabled distinct comparisons between diet-fed and wild bees, providing valuable insights into early microbial acquisition.

Future investigations should precisely elucidate the individual contributions of specific pollen-associated microbes to honey bee physiological parameters, in-hive tasks, and the enhancement of colony defense mechanisms against pathogens. Improvement in sterilisation approaches could also help in confirming the exact influence of the different diets. Furthermore, we particularly recommend exploring pollen-associated microbes as novel probiotics to improve digestion and nutrient absorption, thereby bolstering honey bee health. Additionally, applying advanced ‘omics’ approaches, such as metatranscriptomics and metabolomics, is crucial for a comprehensive understanding of the intricate metabolic activities within this tripartite host-microbe-diet relationship.

## Supplementary information

Below is the link to the electronic supplementary material.

ESM 1 (PDF 275 KB)

## Data Availability

The raw sequence reads used in the present study are available in the Sequence Read Archive Database available in the GenBank database of the NCBI (https://www.ncbi.nlm.nih.gov/sra/PRJNA1180230) under the SRA accession numbers SAMN44517302, SAMN4451730, SAMN44517304, SAMN44517305, and SAMN50704035 with BioProject number PRJNA1180230.
