# Supplementary material for: Dietary modulation of gut microbiota and functional enzymes in savannah honey bees (Apis mellifera scutellata Lepeletier)
Source: Appl Microbiol Biotechnol. 2025 Oct 16;109:226. doi: 10.1007/s00253-025-13615-x (PMC12532740; doi:10.1007/s00253-025-13615-x)
Supplement: Supplementary file 1 [file 253_2025_13615_MOESM1_ESM.pdf]

Applied Microbiology and Biotechnology

Supplementary Material

Dietary modulation of gut microbiota and functional enzymes in savannah honey bees (*Apis mellifera scutellata* Lepeletier)

Nolwandle N. Khumalo[1], Linda U. Obi[1], Abdullahi A. Yusuf[2] and Rasheed A. Adeleke[1*]

[1] Unit of Environmental Sciences and Management, North-West University, Potchefstroom, South Africa
[2] Social Insects Research Group, Department of Zoology and Entomology, University of Pretoria, Hatfield, South Africa

Corresponding author: Rasheed Adeleke, E-mail: Rasheed.adeleke@nwu.ac.za

Table S1 Amplicon Sequence Variant (ASV) table showing unique ASV identifiers and their relative abundance (sequence read counts) and taxonomic classification from phylum to genus across the different honey bee gut treatments

ASV	Casein	Pollen	Sterilised pollen	Sterilised casein	Wild bees	Phylum	Class	Order	Family	Genus
ASV1	0	14	3	0	0	*Pseudomonadota*	*Betaproteobacteria*	*Burkholderiales*	*Alcaligenaceae*	*Achromobacter*
ASV2	3	5	0	0	0	*Actinomycetota*	*Actinomycetes*	*Micromonosporales*	*Micromonosporaceae*	*Actinomarinicola*
ASV3	5	5	0	0	0	*Actinomycetota*	*Actinomycetes*	*Pseudonocardiales*	*Pseudonocardiaceae*	*Actinomycetospora*
ASV4	38	0	0	64	0	*Actinomycetota*	*Actinomycetes*	*Micromonosporales*	*Micromonosporaceae*	*Actinoplanes*
ASV5	79	113	50	48	0	*Actinomycetota*	*Actinomycetes*	*Propionibacteriales*	*Nocardioidaceae*	*Aeromicrobium*
ASV6	0	14	0	0	0	*Actinomycetota*	*Actinomycetes*	*Micrococcales*	*Microbacteriaceae*	*Agromyces*
ASV7	0	0	0	0	2	*Verrucomicrobiota*	*Verrucomicrobiae*	*Verrucomicrobiales*	*Akkermansiaceae*	*Akkermansia*
ASV8	0	0	0	0	846	*Bacteroidota*	*Flavobacteriia*	*Flavobacteriales*	*Flavobacteriaceae*	*Apibacter*
ASV9	14	22	5	18	0	*Bacillota*	*Bacilli*	*Lactobacillales*	*Lactobacillaceae*	*Apilactobacillus*
ASV10	0	5	0	0	0	*Pseudomonadota*	*Alphaproteobacteria*	*Hyphomicrobiales*	*Aurantimonadaceae*	*Aureimonas*
ASV11	4	6	0	0	0	*Pseudomonadota*	*Alphaproteobacteria*	*Rhodospirillales*	*Azospirillaceae*	*Azospirillum*
ASV12	18628	24069	3104	12190	3128	*Pseudomonadota*	*Alphaproteobacteria*	*Hyphomicrobiales*	*Bartonellaceae*	*Bartonella*
ASV13	0	7	0	0	0	*Actinomycetota*	*Actinomycetes*	*Streptosporangiales*	*Streptosporangiaceae*	*Belnapia*
ASV14	2812	1182	3267	1453	11155	*Actinomycetota*	*Actinomycetes*	*Bifidobacteriales*	*Bifidobacteriaceae*	*Bifidobacterium*
ASV15	20	32	0	17	0	*Actinomycetota*	*Actinomycetes*	*Geodermatophilales*	*Geodermatophilaceae*	*Blastococcus*
ASV16	1115	513	271	345	189	*Pseudomonadota*	*Alphaproteobacteria*	*Rhodospirillales*	*Acetobacteraceae*	*Bombella*
ASV17	5	16	8	6	45	*Bacillota*	*Bacilli*	*Lactobacillales*	*Lactobacillaceae*	*Bombilactobacillus*
ASV18	0	53	0	4	0	*Pseudomonadota*	*Alphaproteobacteria*	*Caulobacterales*	*Caulobacteraceae*	*Brevundimonas*
ASV19	244	227	18	283	0	*Actinomycetota*	*Actinomycetes*	*Micrococcales*	*Cellulomonadaceae*	*Cellulomonas*
ASV20	0	4	0	0	0	*Bacteroidota*	*Flavobacteriia*	*Flavobacteriales*	*Flavobacteriaceae*	*Chryseobacterium*
ASV21	4890	4059	4813	3998	14288	*Pseudomonadota*	*Alphaproteobacteria*	*Rhodospirillales*	*Acetobacteraceae*	*Commensalibacter*
ASV22	12	0	0	0	0	*Actinomycetota*	*Actinomycetes*	*Cryptosporangiales*	*Cryptosporangiaceae*	*Cryptosporangium*
ASV23	0	520	4	0	0	*Pseudomonadota*	*Alphaproteobacteria*	*Hyphomicrobiales*	*Devosiaceae*	*Devosia*
ASV24	0	67	0	0	0	*Actinomycetota*	*Actinomycetes*	*Micromonosporales*	*Micromonosporaceae*	*Endobacterium*
ASV25	0	0	0	2	0	*Bacillota*	*Clostridia*	*Oscillospirales*	*Oscillospiraceae*	*Faecalibacterium*
ASV26	0	8	0	0	0	*Bacteroidota*	*Flavobacteriia*	*Flavobacteriales*	*Flavobacteriaceae*	*Flavobacterium*
ASV27	0	0	0	0	33	*Pseudomonadota*	*Gammaproteobacteria*	*Orbales*	*Orbaceae*	*Frischella*
ASV28	0	0	0	2	0	*Pseudomonadota*	*Alphaproteobacteria*	*Rhodospirillales*	*Geminicoccaceae*	*Geminicoccus*
ASV29	0	4	0	10	0	*Actinomycetota*	*Actinomycetes*	*Micrococcales*	*Promicromonosporaceae*	*Isoptericola*
ASV30	0	0	0	0	0	*Actinomycetota*	*Actinomycetes*	*Kineosporiales*	*Kineosporiaceae*	*Kineococcus*

ASV31	79	37	0	74	0	*Actinomycetota*	*Actinomycetes*	*Kineosporiales*	*Kineosporiaceae*	*Kineosporia*
ASV32	0	0	0	6	0	*Actinomycetota*	*Actinomycetes*	*Propionibacteriales*	*Nocardioidaceae*	*Kribbella*
ASV33	145	146	127	210	0	*Bacillota*	*Bacilli*	*Lactobacillales*	*Lactobacillaceae*	*Lactobacillus*
ASV34	75	0	0	30	24	*Pseudomonadota*	*Alphaproteobacteria*	*Hyphomicrobiales*	*Phyllobacteriaceae*	*Mesorhizobium*
ASV35	30	25	19	10	0	*Pseudomonadota*	*Alphaproteobacteria*	*Hyphomicrobiales*	*Methylobacteriaceae*	*Methylobacterium*
ASV36	22	26	0	12	0	*Pseudomonadota*	*Alphaproteobacteria*	*Hyphomicrobiales*	*Methylobacteriaceae*	*Methylorubrum*
ASV37	0	0	0	0	0	*Pseudomonadota*	*Betaproteobacteria*	*Nitrosomonadales*	*Sterolibacteriaceae*	*Methyloversatilis*
ASV38	46	77	0	42	0	*Actinomycetota*	*Actinomycetes*	*Micrococcales*	*Microbacteriaceae*	*Microbacterium*
ASV39	9	0	0	0	0	*Actinomycetota*	*Actinomycetes*	*Propionibacteriales*	*Propionibacteriaceae*	*Microlunatus*
ASV40	0	0	0	32	0	*Actinomycetota*	*Actinomycetes*	*Micromonosporales*	*Micromonosporaceae*	*Micromonospora*
ASV41	0	0	0	0	0	*Pseudomonadota*	*Alphaproteobacteria*	*Hyphomicrobiales*	*Methylobacteriaceae*	*Microvirga*
ASV42	36	35	0	23	0	*Actinomycetota*	*Actinomycetes*	*Geodermatophilales*	*Geodermatophilaceae*	*Modestobacter*
ASV43	7	0	0	4	0	*Actinomycetota*	*Actinomycetes*	*Propionibacteriales*	*Nocardioidaceae*	*Nocardioides*
ASV44	7	20	0	0	0	*Pseudomonadota*	*Alphaproteobacteria*	*Sphingomonadales*	*Sphingomonadaceae*	*Novosphingobium*
ASV45	0	72	0	0	0	*Bacillota*	*Bacilli*	*Caryophanales*	*Paenibacillaceae*	*Paenibacillus*
ASV46	0	76	0	0	0	*Bacteroidota*	*Sphingobacteriia*	*Sphingobacteriales*	*Sphingobacteriaceae*	*Pedobacter*
ASV47	0	3	0	0	0	*Pseudomonadota*	*Gammaproteobacteria*	*Neisseriales*	*Neisseriaceae*	*Pigmentiphaga*
ASV48	123	110	17	111	0	*Actinomycetota*	*Actinomycetes*	*Micrococcales*	*Promicromonosporaceae*	*Promicromonospora*
ASV49	0	0	0	0	0	*Pseudomonadota*	*Gammaproteobacteria*	*Enterobacterales*	*Morganellaceae*	*Providencia*
ASV50	0	49	0	0	0	*Pseudomonadota*	*Alphaproteobacteria*	*Hyphomicrobiales*	*Brucellaceae*	*Pseudochrobactrum*
ASV51	0	0	0	0	0	*Actinomycetota*	*Actinomycetes*	*Micrococcales*	*Micrococcaceae*	*Pseudoglutamicibacter*
ASV52	21	47	70	35	0	*Pseudomonadota*	*Gammaproteobacteria*	*Pseudomonadales*	*Pseudomonadaceae*	*Pseudomonas*
ASV53	0	6	0	0	0	*Actinomycetota*	*Actinomycetes*	*Micrococcales*	*Rhabdothermincolaceae*	*Rhabdothermincola*
ASV54	96	268	17	62	0	*Pseudomonadota*	*Alphaproteobacteria*	*Hyphomicrobiales*	*Rhizobiaceae*	*Rhizobium*
ASV55	97	134	44	63	0	*Actinomycetota*	*Actinomycetes*	*Mycobacteriales*	*Nocardiaceae*	*Rhodococcus*
ASV56	15	31	0	4	0	*Pseudomonadota*	*Alphaproteobacteria*	*Rhodospirillales*	*Acetobacteraceae*	*Roseomonas*
ASV57	0	3	0	0	0	*Actinomycetota*	*Rubrobacteria*	*Rubrobacterales*	*Rubrobacteraceae*	*Rubrobacter*
ASV58	0	61	0	0	0	*Actinomycetota*	*Actinomycetes*	*Micrococcales*	*Sanguibacteraceae*	*Sanguibacter*
ASV59	17	8	0	0	0	*Pseudomonadota*	*Alphaproteobacteria*	*Rhodospirillales*	*Rhodospirillaceae*	*Skermanella*
ASV60	260	139	128	250	282	*Pseudomonadota*	*Betaproteobacteria*	*Neisseriales*	*Neisseriaceae*	*Snodgrassella*
ASV61	0	35	0	0	0	*Pseudomonadota*	*Alphaproteobacteria*	*Sphingomonadales*	*Sphingomonadaceae*	*Sphingomonas*
ASV62	0	19	9	14	0	*Pseudomonadota*	*Gammaproteobacteria*	*Lysobacterales*	*Lysobacteraceae*	*Stenotrophomonas*
ASV63	31	0	2	33	0	*Actinomycetota*	*Actinomycetes*	*Streptomycetales*	*Streptomycetaceae*	*Streptomyces*
ASV64	144	228	110	125	0	*Actinomycetota*	*Actinomycetes*	*Mycobacteriales*	*Nocardiaceae*	*Williamsia*

Table S2 Bray-Curtis dissimilarity values (ranging from 0 to 1) comparing the honey bee gut microbiota across the different treatments. Values closer to 1 indicate a dissimilar bacterial community, while values closer to 0 indicate a similar bacterial composition

	Casein	Pollen	Sterilised pollen	Sterilised casein	Wild bees
**Casein**	0.00	0.16	0.44	0.20	0.53
**Pollen**	0.16	0.00	0.59	0.27	0.60
**Sterilised pollen**	0.44	0.59	0.00	0.41	0.74
**Sterilised casein**	0.20	0.27	0.41	0.00	0.53
**Wild bees**	0.53	0.60	0.75	0.53	0.00

Table S3 Relative abundance (%) at the genus level of the honey bee gut microbiota

	Relative abundance (%)				
**Genus**	**Casein**	**Pollen**	**Sterilised pollen**	**Sterilised casein**	**Wild bees**
Achromobacter	0.00	0.04	0.02	0.00	0.00
Actinoplanes	0.13	0.00	0.00	0.33	0.00
Aeromicrobium	0.27	0.35	0.41	0.25	0.00
Apibacter	0.00	0.00	0.00	0.00	2.82
Apilactobacillus	0.05	0.07	0.04	0.09	0.00
Bartonella	63.95	73.83	25.68	62.26	10.43
Bifidobacterium	9.65	3.63	27,03	7.42	37.19
Blastococcus	0.07	0.10	0.00	0.09	0.00
Bombella	3.83	1.57	2.24	1.76	0.63
Bombilactobacillus	0.02	0.05	0.07	0.03	0.15
Brevundimonas	0.00	0.16	0.00	0.02	0.00
Cellulomonas	0.84	0.70	0.15	1.45	0.00
Commensalibacter	16.79	12.45	39.82	20.42	47.64
Devosia	0.00	1.60	0.03	0.00	0.00
Endobacterium	0.00	0.21	0.00	0.00	0.00
Frischella	0.00	0.00	0.00	0.00	0.11
Kineosporia	0.27	0.11	0.00	0.38	0.00
Lactobacillus	0.50	0.45	1.05	1.07	0.00
Methylobacterium	0.10	0.08	0.16	0.05	0.00
Methylorubrum	0.08	0.08	0.00	0.06	0.00
Microbacterium	0.16	0.24	0.00	0.21	0.00
Modestobacter	0.12	0.11	0.00	0.12	0.00
Novosphingobium	0.02	0.06	0.00	0.00	0.00
Pedobacter	0.00	0.23	0.00	0.00	0.00
Promicromonospora	0.42	0.34	0.14	0.57	0.00
Pseudomonas	0.07	0.14	0.58	0.18	0.00
Rhizobium	0.33	0.82	0.14	0.32	0.00
Rhodococcus	0.33	0.41	0.36	0.32	0.00

Roseomonas	0.05	0.10	0.00	0.02	0.00
Sanguibacter	0.00	0.19	0.00	0.00	0.00
Snodgrassella	0.89	0.43	1.06	1.28	0.94
Streptomyces	0.11	0.00	0.02	0.17	0.00
Williamsia	0.49	0.70	0.91	0.64	0.00

Table S4 The standardized Z-values comparing the gut microbial community across the different treatments

Genus	Casein	Pollen	Sterilised pollen	Sterilised casein	Wild bees
Apibacter	-0.44	-0.44	-0.44	-0.44	1.78
Actinoplanes	0.59	-0.69	-0.69	1.48	-0.69
Aeromonas	-0.44	-0.44	-0.44	-0.44	1.78
Aeromicrobium	0.54	1.29	-0.23	-0.21	-1.38
Bartonella	0.56	1.25	-1.41	-0.25	-0.14
Bifidobacterium	-0.38	-0.53	-0.34	-0.51	1.78
Blastococcus	0.27	1.47	-0.92	0.10	-0.92
Bombella	1.69	0.07	-0.58	-0.38	-0.80
Bombilactobacillus	-0.44	-0.44	-0.44	-0.44	1.78
Brevundimonas	-0.44	1.78	-0.44	-0.44	-0.44
Cellulomonas	0.66	0.53	-1.04	0.96	-1.11
Commensalibacter	0.65	0.25	0.61	0.22	-1.75
Devosia	-0.44	1.78	-0.44	-0.44	-0.44
Endobacterium	-0.44	1.78	-0.44	-0.44	-0.44
Kineosporia	0.40	-0.54	-1.38	0.28	1.23
Lactobacillus	0.62	0.15	-0.53	1.15	-1.40
Methylobacterium	1.31	0.71	-0.92	-0.17	-0.92
Methylorubrum	0.88	1.21	-0.91	-0.26	-0.91
Microbacterium	0.39	1.45	-0.93	0.01	-0.93
Modestobacter	0.96	0.90	-1.04	0.23	-1.04
Novosphingobium	-0.44	1.78	-0.44	-0.44	-0.44
Pedobacter	-0.44	1.78	-0.44	-0.44	-0.44
Promicromonospora	0.86	0.56	-0.93	0.72	-1.23
Pseudochrobactrum	-0.44	1.78	-0.44	-0.44	-0.44
Pseudomonas	-0.44	-0.44	-0.44	1.78	-0.44
Rhizobium	0.00	1.70	-0.59	-0.30	-0.81
Rhodococcus	0.51	1.30	-0.43	-0.02	-1.36
Roseomonas	0.56	1.48	-0.80	-0.43	-0.80
Sanguibacter	-0.52	-0.19	-0.52	-0.52	1.77
Snodgrassella	0.92	-0.08	-0.45	1.00	-1.38
Streptomyces	1.12	-0.73	-0.73	1.06	-0.73
Williamsia	0.28	1.30	-0.16	0.05	-1.47

Table S5 Enzymes predicted to be involved in the digestion and nutrition processes across the different honey bee gut treatments

Description	Casein	Pollen	Sterilised pollen	Sterilised casein	Wild bees
β-Galactosidase (EC:3.2.1.23)	9518.76	12868.83	8805.28	6835.8	38243.54
Subtilisin (EC: 3.4.21.62)	32	244	29	21	0
Triacylglycerol lipase (EC:3.1.1.3)	680	1533	435	392.8	48
Cellulose 1,4-beta-cellobiosidase (EC:3.2.1.91)	284.63	179.86	30.29	287.85	2
Cellulase (EC: 3.2.1.4)	2026.09	6200.68	1480.69	1746.19	10049.5
β-Glucosidase (EC:3.2.1.21)	22250.68	26108.88	22160.94	39912.07	63443.54
α-Amylase (EC:3.2.1.1)	3610.25	7299.18	3154.49	27695.39	486.78
β-Fructofuranosidase (EC:3.2.1.26)	5790.84	4088	6720.66	3351.6	20216.5
3-Phytase (EC: 3.1.3.8)	180	347	26	139.8	0

Table S6 Enzymes predicted to be involved in carbohydrate metabolism across the different honey bee gut treatments

Description	Casein	Pollen	Sterilised pollen	Sterilised casein	Wild bees
Cellulase (EC:3.2.1.4)	2026.09	6200.68	1480.69	1746.19	10049.5
β-Glucosidase (EC:3.2.1.21)	22250.68	26108.88	22160.94	39912.07	57776.75
Glucokinase (EC:2.7.1.2)	14690.14	25453.98	6753.74	35232	7516.04
Glucose-6-phosphate isomerase (EC:5.3.1.9)	18934.26	24894.4	12448.87	37463.07	21153.54
Malate dehydrogenase (EC:1.1.1.37)	11794.51	19330.65	4459.12	32977.69	8275.29
Isocitrate dehydrogenase (EC:1.1.1.41)	3719.5	3466	4301.5	2452.5	2746
Pyruvate dehydrogenase (EC:1.2.4.1)	43563.78	62365.51	29165.81	105047.15	31326.29
Oxoglutarate dehydrogenase (EC:1.2.4.2)	14837.45	21663.32	7573.62	10250.99	11009.29
Glyceraldehyde-3-phosphate dehydrogenase (EC:1.2.1.12)	19721.16	26229.4	12815.99	38202.73	21291.87
Fumarate hydratase (EC:4.2.1.2)	19802.19	29983.88	14685.74	38659.11	14041.25
Succinate-CoA ligase (EC:6.2.1.5)	28353.9	40081.64	13591.24	18763.48	36633.08
Aconitate hydratase (EC:4.2.1.3)	18845.41	25910.82	12535.12	37335.09	21896.33
Citrate (Si)-synthase (EC:2.3.3.1)	20801.21	29384.58	13216.65	38842.75	24880.5
Pyruvate kinase (EC:2.7.1.40)	19646.39	25744.1	13602.16	62697.87	21162
Phosphopyruvate hydratase (EC:4.2.1.11)	19063.99	24858.93	12429.16	37557.42	21153.54
Phosphoglycerate mutase (EC:5.4.2.12)	34572.97	41829.7	27701.19	98371.82	41487.79
Phosphoglycerate kinase (EC:2.7.2.3)	18755.7	24571.07	12350.87	37301.99	21153.54
Triose-phosphate isomerase (EC:5.3.1.1)	19198.47	26094.79	12582.66	37648.9	24128.54
Fructose-bisphosphate aldolase (EC:4.1.2.13)	20696	27967.68	14003.91	63811.12	25188.54
